# Perceptual Priming Can Increase or Decrease With Aging

**DOI:** 10.3389/fnagi.2020.576922

**Published:** 2020-11-19

**Authors:** Kalathupiriyan A. Zhivago, Sneha Shashidhara, Ranjini Garani, Simran Purokayastha, Naren P. Rao, Aditya Murthy, S. P. Arun

**Affiliations:** ^1^Centre for Neuroscience, Indian Institute of Science, Bengaluru, India; ^2^Department of Psychiatry, National Institute of Mental Health and Neurosciences, Bengaluru, India

**Keywords:** perception, priming, implicit memory, perceptual priming, visual search, aging, mild cognitive impairment

## Abstract

A decline in declarative or explicit memory has been extensively characterized in cognitive aging and is a hallmark of cognitive impairments. However, whether and how implicit perceptual memory varies with aging or cognitive impairment is unclear. Here, we compared implicit perceptual memory and explicit memory measures in three groups of participants: (1) 59 healthy young volunteers (20–30 years); (2) 269 healthy old volunteers (50–90 years) and (3) 21 patients with mild cognitive impairment, i.e., MCI (50–90 years). To measure explicit memory, participants were tested on standard recognition and recall tasks. To measure implicit perceptual memory, we used a classic perceptual priming paradigm. Participants had to report the shape of a visual search pop-out target whose color or position was varied randomly across trials. Perceptual priming was measured as the speedup in response time for targets that repeated in color or position. Our main findings are as follows: (1) Explicit memory was weaker in old compared to young participants, and in MCI patients compared to age- and education-matched controls; (2) Surprisingly, perceptual priming did not always decline with age: color priming was smaller in older participants but position priming was larger; (3) Position priming was less frequent in the MCI group compared to matched controls; (4) Perceptual priming and explicit memory were uncorrelated across participants. Thus, perceptual priming can increase or decrease with age or cognitive impairment, but these changes do not covary with explicit memory.

## Introduction

Memory has broadly been classified into explicit and implicit memory (Squire, 1992; Schacter et al., 1993; Gazzaniga et al., 2014). Explicit memory is consciously accessible and declarative; it is measured by how well participants can recall items that were previously studied (Strauss et al., 2006). By contrast, implicit memory is unconscious and non-declarative; it is measured by the facilitation in the response to previously experienced items (Schacter et al., 1993; Fleischman et al., 2005; Spataro et al., 2016). Since a decline in explicit memory is a hallmark of both aging and cognitive disorders, the question of whether implicit memory is also affected has been extensively investigated (Mitchell and Bruss, 2003; Fleischman, 2007; Berry et al., 2008; Ward et al., 2013). In most studies, implicit memory is measured as an increased probability of producing a studied item in an unrelated task, or by the facilitated recognition of a fragmented picture after it was previously viewed. The results are mixed: explicit memory always shows a clear decline with age and cognitive impairments, but implicit memory declines in some cases (Perri et al., 2007; Soldan et al., 2009; Ballesteros et al., 2013; Gordon et al., 2013; Boccia et al., 2014) but not others (Fleischman et al., 2005; LaVoie and Faulkner, 2008). Nonetheless it has been proposed that an implicit memory deficit could be an early sign for the onset of dementia (Fleischman, 2007).

Despite these insights, the commonly used implicit memory tests have several problems. First, participants may use explicit memory during the study phase. This “explicit contamination” can be mitigated but is extremely tricky to fully rule out (Fleischman, 2007). Second, these tests assume a minimum proficiency in verbal and object naming which may vary widely especially in diverse populations with varying degrees of multilingualism and literacy.

One potential solution to these issues is to develop tasks that are culture-free with no study phase. Recent studies have addressed this issue by measuring the facilitation in categorical responses upon repeated viewing of objects, and have shown that this priming is weaker for older participants but only for unfamiliar objects (Soldan et al., 2009; Gordon et al., 2013). While these tasks require processing complex object properties, they have the advantage that they enable the comparison of implicit and explicit memory for the same items.

Here, we devised an implicit memory paradigm based on a classic perceptual priming effect, known as priming of pop-out (Maljkovic and Nakayama, 1994, 1996). In this paradigm, participants are faster to respond to the shape of a pop-out target when its color or position is repeated. This task has several advantages over implicit verbal memory measures. First, the task is easy to comprehend and assumes no prior knowledge of objects or words, making it suitable for use on diverse populations. Second, the task does not involve a study phase, thereby mitigating any explicit contamination. Third, since participants have to report the target shape while its color and position are manipulated independently, the speedup in their responses due to making repeated responses (i.e., motor priming) can be decoupled from any effects of repeated target color or position. However, this task suffers from having very little variation in item shape, with the result that explicit and implicit memory cannot be compared for the same items (Schacter et al., 1993; Constantinidou and Baker, 2002). Although priming of pop-out is a well-known paradigm, how this effect varies across age groups or across cognitive disorders and whether it covaries with explicit memory has never been investigated previously. Our goal therefore was to characterize how this particular form of implicit perceptual memory varies across age and cognitive impairments and assess whether it covaries with explicit memory measures.

## Materials and Methods

All participants had normal or corrected-to-normal vision and gave written informed consent to an experimental protocol approved by the Institutional Human Ethics Committee of the Indian Institute of Science, Bangalore. All experiments were conducted in accordance with the relevant guidelines and regulations. All participants were compensated monetarily for their participation.

### Participants

Young volunteers were all students from the Indian Institute of Science campus. Older volunteers were all from an urban, literate background and recruited through extensive community engagement, and were all participants of an ongoing Tata Longitudinal Study of Aging (TLSA). Older volunteers underwent standard clinical and neuropsychological evaluations, based on which they were labeled as healthy or MCI (McKhann et al., 2011). The experimenters performing this study were blind to these labels during data collection and were given the label only afterward for the purposes of analysis. The older volunteers were larger in number since they were part of an ongoing study, whereas the younger volunteers were recruited for the purposes of the age comparisons in this study.

In all, we analyzed data from 59 young participants (25 ± 3 years, 29 female; years of education: 19 ± 3 years), 269 old participants (67 ± 8 years, 119 female; years of education: 19 ± 3 years), and 21 MCI patients (72 ± 10 years, 4 female; years of education: 15 ± 5 years). From these older participants, we selected a subset of participants whose age was similar to the MCI patients, and with similar number of years of education. This age- and education-matched control group (hereafter referred to as matched controls) comprised 92 participants (70 ± 10 years, 45 female; years of education: 16 ± 2 years).

### MCI Diagnosis

The Clinical Dementia rating (CDR) scale (Hughes et al., 1982) was administered by trained research staff and a diagnosis was given based upon the scores to all the TLSA participants. CDR is administered by interviewing the volunteer and their primary caregiver (typically a close relative), which took about 20–30 min. A score of 0 corresponded to a healthy volunteer and 0.5 to MCI. MCI patients identified among the TLSA participants were broadly classified as amnestic or non-amnestic type. All the MCI patients (*n* = 21) in this study were of the amnestic type.

### Global Cognitive Scores

To validate the MCI Diagnosis obtained from the CDR scale, we also compared the global cognitive score ACE III (Hsieh et al., 2013), measured from the older volunteers as part of the Tata Longitudinal Study of Aging. As expected, ACE-III scores were significantly higher for matched controls compared to MCI patients (ACE-III score, mean ± std: 94 ± 4 for matched controls, 87 ± 7 for MCI patients, cohen’s d = 1.22, *p* < 0.00005, rank-sum test).

### Procedure

All tasks were administered through HTML5/Javascript scripts run on an internet browser on a desktop computer (24-inch, width x height: 53.3 × 30.0 cm, 1920 × 1080 pixels) or laptop computer (15.4-inch, width × height: 33.0 × 20.6 cm, 2880 × 1800 pixels). All displayed items were scaled using the monitor size and viewing distance so that they subtended the same visual angle. The browser-based setup was validated by comparing it with visual search tasks written in Matlab and Psychtoolbox. Each participant performed a perceptual priming task, an object recognition task, a word recall and word recognition task in that order, as detailed below.

### Perceptual Priming Task

**FIGURE 1:**
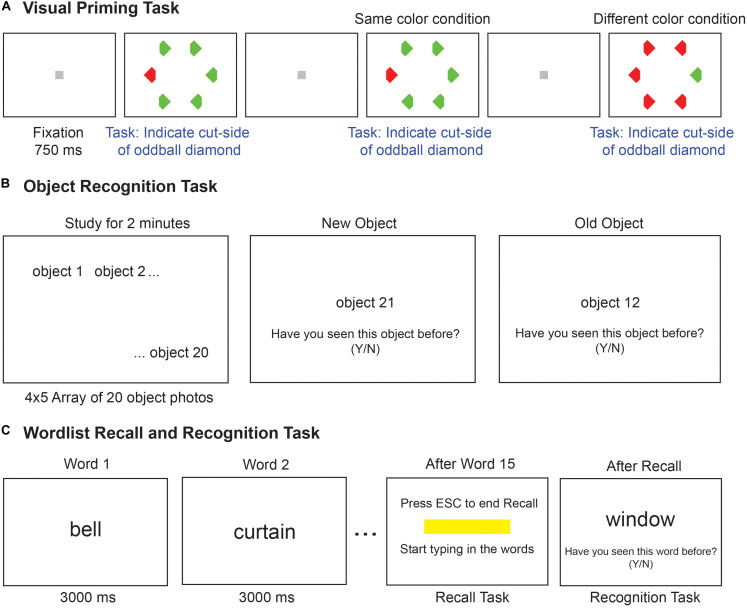
Implicit and explicit memory tasks. **(A)** Schematic of the visual priming task with its two possible conditions. Each trial started with a gray fixation square followed by a search array. The second trial shows the odd–colored target with the same color (*red*) as the previous target, hence it is the same-color condition. The next trial shows the case where the target color (*green*) is different from the previous trial (*red*), hence it is the different-color condition. Participants make faster responses on same-color trials compared to different-color trials, indicative of an implicit memory. **(B)** Schematic of the object recognition task. Participants were asked to study a set of 20 common object photographs for 2 minutes. In the test phase (second and third panels), one picture was shown at a time and participants had to indicate whether the object was shown or not shown during the study phase. **(C)** Schematic of the word recall and recognition tasks. During the study phase (*left panel*), 15 words are presented for 3 s each in a sequence. In the recall phase (*middle panel*), a text box appeared on the screen, and the experimenter typed in the words recalled verbally by the participant. In the recognition task (*right panel*), 30 words were presented in sequence, and participants had to indicate whether the word was shown or not during the study phase.

Each trial started with a gray fixation square (0.4° × 0.4°) displayed for 750 ms at the center of the screen, followed by a hexagonal search array (with items placed 6° from the center) containing one oddball colored item among five other distractors (Figure 1A). Each target or distractor shape was a diamond measuring 2° along the longer dimension with a 1° vertical cut on the left or right side. The array comprised a red target among green distractors or vice-versa, with the target chosen to appear randomly either at the leftmost or the rightmost location. Participants were instructed to indicate whether the oddball target diamond was cut on the right or left side by pressing the corresponding arrow key on a keyboard, and were asked to respond as quickly and accurately as possible. The search array was displayed for 10 s, or until the participant made a valid response, whichever was shorter. Target color and position were counterbalanced (i.e., equal numbers of red/green x left/right trials) and presented in random order. Error trials and trials with no response were repeated later after a random number of other trials. Participants performed a total of 200 correct trials.

Priming strength was calculated as the percent decrease in response time on a trial preceded by a target of the same color/position relative to the response time on trials where the target was of a different color/position. Thus,

$$primingstrength=100^{*}\left(DRT-SRT\right)/DRT$$

where, *DRT* is the mean reaction time of trials preceded by a different color/position compared to the current trial and *SRT* is the mean reaction time of trials preceded by the same color/position compared to the current trial. In the analyses reported here, we calculated priming strength using only consecutive trials with correct responses, but we obtained qualitatively similar results upon using all trials regardless of error status. We also obtained qualitatively similar results on using SRT in the denominator instead of DRT.

Since the trials were presented in random order, the different groups of participants might have slightly different numbers of trials that entered the SRT and DRT calculations. However, this was not the case: all groups had similar numbers of trials (mean ± sd of number of trials for SRT and DRT: 94 ± 9 and 90 ± 10 for young; 95 ± 8 and 92 ± 8 for old; 92 ± 10 and 92 ± 9 for MCI patients; 96 ± 8 and 91 ± 8 for matched controls.

### Object Recognition Task

Each participant was asked to study a total of 20 objects (measuring 3.5° along the longer dimension) arranged in a 4 × 5 grid for 2 min, and were informed that they would be tested on these objects afterward. These were pictures of common objects such as animals, household objects, vehicles, etc., (Figure 1B). After the study phase, 40 pictures were presented, one at a time, prompting the participant to press ‘y’ or ‘n’ key to indicate whether (s)he saw the object during the study phase or not, with no time restriction on each response. Half of these were old (i.e., from the study phase) and the remaining half were new. Each new image was from the same basic-level category as the corresponding old image. The participants were instructed that they do not have to name any picture or for that matter give any descriptive account. Object recognition memory performance was characterized for each participant by calculating the total percentage correct across old and new items.

### Word Recall and Recognition Tasks

Each participant performed a word recall task and a recognition task (McMinn et al., 1988), always in this order (Figure 1C). The word recall task started with a study phase in which 15 words (e.g., color, garden, coffee, house, etc.) were presented on the screen in a predefined sequence, with each word shown for 3 s. This was followed by a recall phase in which the participants were asked to recall as many words as they could from the study phase, and the experimenter typed in the words. Participants were free to recall words in any order and take any amount of time. The task was stopped once the participant declared that they could not remember any more words. Word recall performance was calculated for each participant as the fraction of words correctly recalled out of the full list.

During the recognition task, one word was presented at a time and the participant was asked to report with a key press, if the word was presented in the recall block (‘y’ for yes and ‘n’ for no). A total of 30 words were presented, 15 of which belonged to the recall block and 15 were new. The new words were drawn from similar categories as the study words (e.g., crayon, tree, home, etc.). Words did not overlap in content with the objects used in the object recognition task. Word recognition performance was calculated for each participant as the fraction of old and new words that elicited a correct response. Because our cohort was mostly urban and literate, all participants were assumed to be familiar with the words being shown, so it is unlikely that word novelty would affect recall or recognition. We also did not find any systematic relation between the number of years of education and the explicit memory measures.

### Statistical Testing

Since accuracy and response times are frequently non-normal, we used non-parametric tests to compare participant groups (Wilcoxon signed-rank test for paired comparisons and Wilcoxon rank-sum test for unpaired comparisons). We used ANOVA for multifactorial comparisons since there are no non-parametric analogs. Both these tests take into account the unequal sample sizes across groups. Using parametric or nonparametric tests yielded qualitatively similar results.

### Calculation of d’ measure

We calculated a measure of discrimination (d’) and bias (C) for participant scores on the word recognition and object recognition tasks (Snodgrass and Corwin, 1988). In each task, we calculated the fraction of correct responses to old objects out of all old object responses as hits (H), and the fraction of incorrect responses to new objects out of all new object responses as false alarms (FA). To avoid infinite values arising from H = 1 or FA = 0, we reduced these numbers by 1/N where N is the total number of trials (*N* = 40 for object recognition, *N* = 30 for word recognition tasks). The d’ measure was then calculated as *d*′ = *z*(*H*)−*z*(*FA*), where the function *z*(*x*) refers to the inverse cumulative distribution function of a standard normal distribution at the value *x*. The bias measure was calculated as *C* = −(*z*(*H*) + *z*(*FA*))/2. A positive bias implies that participants avoided false alarms at the expense of misses (i.e., avoided declaring new items as old).

## Results

Our goal was to characterize explicit and implicit memory across age and cognitive impairments. We compared these measures between young vs older volunteers and between MCI patients and age- and education-matched controls. The tasks performed by each participant are detailed in Figure 1.

In the implicit perceptual priming task (Figure 1A), participants had to report the shape of an oddball item in a hexagonal search array while its position or color was varied independently. As a result, any response speedup due to repeated color or position is independent of influences from making repeated motor responses (i.e., independent of motor priming).

We compared the performance on this implicit perceptual priming task with three explicit memory tasks. In the object recognition task (Figure 1B), participants were asked to study an array of objects and had to discriminate studied items from novel items. We selected this task because it is a measure of explicit visual object memory as opposed to verbal memory. In the word recall and recognition tasks (Figure 1C), participants were shown a series of words presented on a monitor for 3 s each, and were subsequently asked to recall these words (word recall task) or discriminate them from novel words (word recognition task).

### Perceptual Priming Task

All four groups of participants were highly accurate on this task (accuracy, mean ± sd: 96 ± 5% for young, 97 ± 4% for old; 97 ± 4% for matched controls, and 95 ± 7% for MCI patients). Compared to young participants, older participants were generally more accurate (cohen’s d = 0.4, z = 3.85, *p* < 0.0005, rank-sum test on overall accuracy for young vs. old), but were considerably slower (average RT, mean ± sd: 1.02 ± 0.34 s for young; 1.75 ± 0.56 s for old; cohen’s d = 1.2, z = 9.66, *p* < 0.00005, rank-sum test). By contrast, compared to matched controls, patients were less accurate (z = 2.38, *p* = 0.02, rank-sum test) but equally fast (mean ± sd of RT: 1.97 ± 0.62 s, 1.86 ± 0.71 s, cohen’s d = 0.16, z = 1.17, *p* = 0.24, rank-sum test). Thus, MCI patients slightly less accurate but similar in speed compared to matched controls.

**FIGURE 2:**
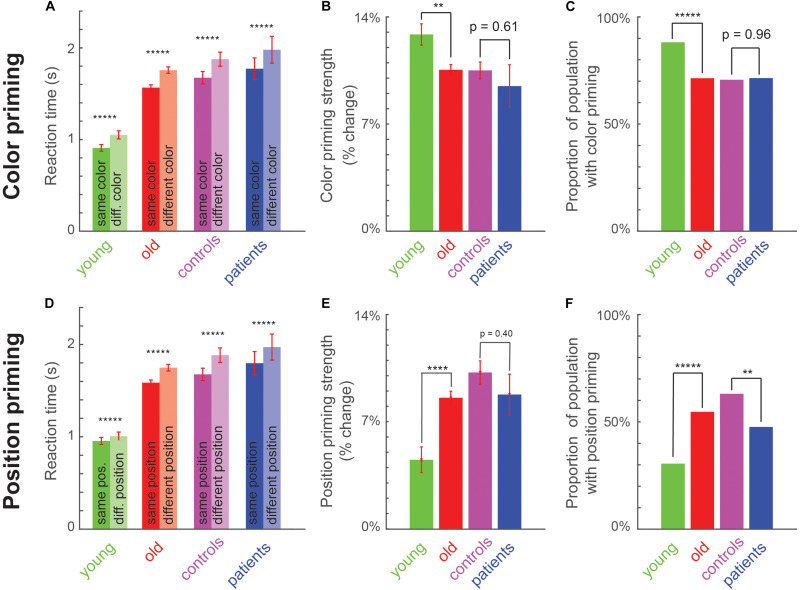
Perceptual priming effects across age and cognitive impairments. **(A)** Average reaction time for same-color (dark) and different-color (light) trials across young, old, matched controls (am old) and patients. Asterisks indicate statistical significance of the main effect of color in an ANOVA (Supplementary Section S2): * is *p* < 0.05, ** is *p* < 0.005, etc., Error bars indicate SEM across participants. **(B)** Color priming strength across groups. Asterisks indicate statistical significance comparing priming strength across participants between groups, using a rank-sum test (*p < 0.05, **p < 0.005, etc.). Error bars indicate SEM across participants. **(C)** Proportion of population with significant color priming shown for each group of participants. Asterisks indicate statistical significance comparing the rate of incidence between each pair of groups, using a chi-squared test, with conventions as before. **(D–F)** Same as **(A–C)** but for position priming.

To measure implicit memory, we compared the reaction time on trials preceded by a target of the same versus different color or position. In all four groups (young, old, matched controls and patients), participants were faster for same-color trials compared to different-color trials in all groups (Figure 2A), and were faster for same-position trials compared to different-position trials (Figure 2D). A detailed statistical comparison is provided in Supplementary Section S1.

Participants were also more accurate on the same-color trials compared to different-color trials in all groups (accuracy, mean ± sem for same and different color trials: 97% ± 0.6% and 93% ± 1% for young participants; 97.8% ± 0.2% and 96.5% ± 0.3% for older participants; 94.9% ± 1.5% and 94.5% ± 1.6% for MCI patients; 97.6% ± 0.3% and 96.4% ± 0.5% for matched controls). Likewise, participants were more accurate on same-position trials compared to different position trials for each group (accuracy, mean ± sem for same and different position trials: 97.3% ± 0.6% and 94.9% ± 0.7% for young; 98% ± 0.2% and 96.3% ± 0.3% for older participants; 95.4% ± 1.6% and 93.9% ± 1.6% for MCI patients; 97.8% ± 0.4% and 96.2% ± 0.5% for matched controls). Thus, the faster responses in the same-color and same-position trials cannot be explained as a speed accuracy tradeoff.

Thus, priming of pop-out is robustly present at the group level in young, old, matched controls and patients.

### Do Color and Position Priming Vary With Age or With Cognitive Impairment?

Next we asked whether the strength of priming was different across age or with cognitive impairment. To this end, we calculated the priming strength for each participant as the percentage change in reaction time between primed (i.e., same color or position) and unprimed (different color or position) trials. Calculating the percentage change ensures that the measure is normalized to the speed of each participant and therefore comparable across participant groups.

To ascertain the reliability of variations in priming strength across participants, we calculated the priming strength using odd and even-numbered repetitions for each participant, and asked whether the two measures were correlated across participants. This split-half correlation, which is an index of reliability, was moderate in magnitude and statistically significant for both color and position priming strengths (r = 0.43 for color priming and 0.55 for position priming, *p* < 0.00005 in both cases; calculated across the older group). However, we note that these correlations do not reflect the correlation between participants that would be obtained on repeating the entire experiment (i.e., the test-retest reliability), since they are based on comparing two halves of the data. To estimate the expected test-retest correlation, we performed a Spearman-Brown correction given by *rc* = 2*r*/(1 + *r*), where r is the split-half correlation. These estimated test-retest correlations were large in both cases, suggesting that the priming strength measures are robust across participants (r = 0.60 and 0.71 for color and position priming).

Next we examined differences in color and priming strengths across groups. Color priming strength was significantly weaker for older participants compared to young participants (Figure 2B; Priming strength: 13 ± 1% for young, 11 ± 0% for old, cohen’s d = 0.41, z = 2.77, *p* < 0.05, rank-sum test). It was numerically weaker for patients compared to matched controls, but this effect was not statistically significant (Figure 2B; Priming strength: 9 ± 1% for patients, 11 ± 1% for matched controls, cohen’s d = 0.19, z = 0.51, *p* = 0.61, rank-sum test). By contrast, position priming was stronger in older participants compared to young (Figure 2E; Priming strength: 5 ± 1% for young, 8 ± 0% for old, cohen’s d = 0.56, z = 4.25, *p* < 0.0005, rank-sum test). As with color priming, position priming was numerically weaker in patients compared to matched controls but this effect was not statistically significant (Priming strength: 9.1 ± 0.7% for patients, 10 ± 1% for matched controls, cohen’s d = 0.2, z = 0.89, *p* = 0.37, rank-sum test).

The variations in color and priming strength between young and old participants may indicate a general change in priming strength with age, or a selective change in a subset of participants. To explore these possibilities, we performed a participant-wise analysis to detect the presence of color or position priming. For each participant, we performed an ANOVA on the response times, with color, position and motor priming as factors. The fraction of participants with a significant color priming effect in each group is shown in Figure 2C. Color priming was less prevalent in old compared to young participants (Figure 2C; Percentage of participants with significant color priming: 88% for young, 71% for old, χ^2^ = 70.67, *p* < 0.000005, chi-squared test comparing young participants with and without color priming against the numbers predicted using the incidence in the smaller group, i.e., young participants; χ^2^ = 7.31, *p* = 0.007 for the same test with incidence predicted using the larger group). It was also less prevalent among patients compared to matched controls, but this difference was not statistically significant (Figure 2C; Percentage of participants with significant color priming: 71.4% for patients, 70.6% for matched controls, χ^2^ = 0.03, *p* = 0.87, chi-squared test using incidence predicted from smaller group; χ^2^ = 0.002, *p* = 0.96).

By contrast, position priming was more prevalent among old compared to young participants (Figure 2F; Percent of participants with significant position priming: 31% for young, 54% for old, χ^2^ = 73, *p* < 0.000005, chi-squared test using incidence predicted from the smaller group; χ^2^ = 12.9, *p* < 0.0005 using the larger group). It was also significantly less prevalent among MCI compared to matched controls (Figure 2F; Percentage of participants with significant position priming: 48% for patients, 62% for matched controls, χ^2^ = 8.2, *p* = 0.0043, chi-squared test using smaller group; χ^2^ = 1.53, *p* = 0.22 using the larger group). We obtained qualitatively similar results upon selecting participants with position priming strength above a threshold.

Finally, we asked whether participants exhibited differences in motor priming, i.e., whether they were faster when they had to make the same motor response (indicating the cut-side of the diamond) on consecutive trials, compared to when they had to make a different motor response. Motor priming did not differ in strength between young and old participants (priming strength: -1 ± 1% for young, 0 ± 0% for old, cohen’s d = 0.24, z = 1.5, *p* = 0.09, rank-sum test) or between patients and matched controls (priming strength: 0 ± 1% for patients, 0 ± 0% for matched controls, cohen’s d = 0.13, z = 0.20, *p* = 0.84, rank-sum test). But its incidence was relatively low across participants and decreased with age (percent of participants with significant motor priming: 14% in young, 7% in old, χ^2^ = 9.1, *p* < 0.005, chi-squared test using incidence from smaller group; χ^2^ = 2.9, *p* = 0.09 using larger group). Motor priming was numerically less frequent in patients compared to controls but this trend was not statistically significant (percent of participants with significant motor priming: 0% in patients, 8.7% in matched controls, χ^2^ = 2.3, *p* = 0.13, chi-squared test using smaller group; χ^2^ = 0.06, *p* = 0.8 using larger group; assuming non-zero prevalence in patients). Thus, motor priming is weakly present in general but declines across age.

In sum, we conclude that implicit perceptual priming for color and position show differential effects across age: Older participants show weaker color and motor priming but stronger position priming compared to young subjects. Position priming was less prevalent among MCI patients compared to matched controls.

### Explicit Memory Differences

The word recall and recognition tasks used in our study involved making computer-based responses as well as viewing study words visually and recalling them verbally. To be sure that performance on these tasks is similar to the more standard verbally administered word recall and recognition tasks, we asked whether participants performance was correlated between our tasks and an independently administered word recall and recognition task from the Tata Longitudinal Study of Aging, in which an independent word list was used and words were presented purely verbally. This revealed a significant positive correlation (correlation coefficient across older volunteers: immediate word recall: r = 0.37, *p* < 0.000005 and immediate word recognition: r = 0.15, *p* = 0.052). The relatively low correlation may be due to the difference in presentation versus test modality in our task (Schacter, 1992; Schacter et al., 1993).

**FIGURE 3:**
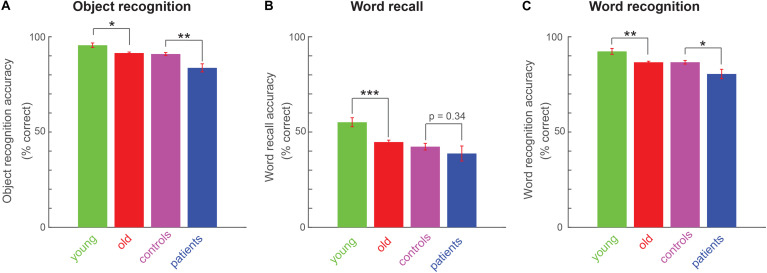
Explicit memory variations across age and cognitive impairments. **(A)** Object recognition accuracy (percentage correct) across all participant groups. Asterisks indicate statistical significance comparing participant-wise accuracy using a rank-sum test. Conventions are as before. **(B)** Word recall performance (percentage correct) across all four participant groups, with conventions as in **(A)**. **(C)** Word recognition performance (percentage correct) across all four participant groups, with conventions as in **(A)**.

Next we investigated whether explicit memory differs across age and cognitive impairments. Object recognition memory was significantly weaker in old compared to young participants (Figure 3A; average accuracy: 92 ± 1% for old, 96 ± 1 for young, cohen’s d = 0.49, z = 2.62, *p* < 0.05, rank-sum test) and in patients compared to matched controls (Figure 3A; average accuracy: 84 ± 1% for patients, 91 ± 1 for matched controls, cohen’s d = 0.89, z = 3.31, *p* < 0.005, rank-sum test).

Likewise, word recall was significantly worse for old compared to young participants (Figure 3B; average percentage of words recalled: 45 ± 1% for old, 55 ± 2 for young, cohen’s d = 0.6, z = 3.91, *p* < 0.0005, rank-sum test). Patients showed weaker word recall compared to matched controls, but this effect was not significant (average percentage of words recalled: 39 ± 4% for patients, 42 ± 2 for matched controls, cohen’s d = 0.21, z = 0.95, *p* = 0.34, rank-sum test).

Finally, word recognition was weaker for old compared to young participants (Figure 3C; average accuracy on old/new word recognition: 87 ± 1% for old, 92 ± 2 for young, cohen’s d = 0.63, z = 2.91, *p* < 0.005, rank-sum test). Patients showed significantly worse word recognition compared to controls (Figure 3C; average accuracy: 80 ± 2% for patients, 87 ± 1 for matched controls, cohen’s d = 0.63, z = 2.45, *p* < 0.05, rank-sum test).

To confirm that the same trends are present using other performance measures, we calculated a d’ measure of performance for the two recognition tasks, as well as their response bias (see Methods). In the object recognition task, older participants had a significantly smaller d’ compared to younger participants (d’, mean ± sem: 3.7 ± 0.2 for young, 3.28 ± 0.17 for old, cohen’s d = 0.52, z = 2.43, *p* < 0.05, rank-sum test), and patients had smaller d’ compared to matched controls (d’, mean ± sem: 2.27 ± 0.08 for patients, 3.08 ± 0.14 for matched controls, cohen’s d = 0.87, z = 3.6, *p* < 0.0005, rank-sum test). However, we observed no systematic differences in response bias (C, mean ± sem: −0.09 ± 0.04 for young, −0.03 ± 0.02 for old, cohen’s d = 0.15, z = 0.92, *p* = 0.36, rank-sum test; −0.04 ± 0.08 for patients, −0.02 ± 0.04 for matched controls; cohen’s d = 0.04, z = 0.4, *p* = 0.69).

In the word recognition task, older participants had significantly smaller d’ compared to young participants (d’, mean ± sem: 1.66 ± 0.1 for young, 1.39 ± 0.03 for old, cohen’s d = 0.55, z = 2.6, *p* = 0.009) and patients had smaller d’ than matched controls (d’, mean ± sem: 1.15 ± 0.09 for patients, 1.39 ± 0.05 for controls, cohen’s d = 0.5, z = 2, *p* = 0.042). However, we observed no systematic difference in response bias (C, mean ± sem: 0.89 ± 0.04 for young, 0.83 ± 0.01 for old, cohen’s d = 0.24, z = 1.2, *p* = 0.22; 0.83 ± 0.07 for patients, 0.83 ± 0.03 for controls, cohen’s d = 0.01, z = 0.2, *p* = 0.84).

We conclude that explicit memory measures are weaker in older participants compared to younger participants, and in patients compared to controls.

### Relation Between Implicit and Explicit Memory

**FIGURE 4:**
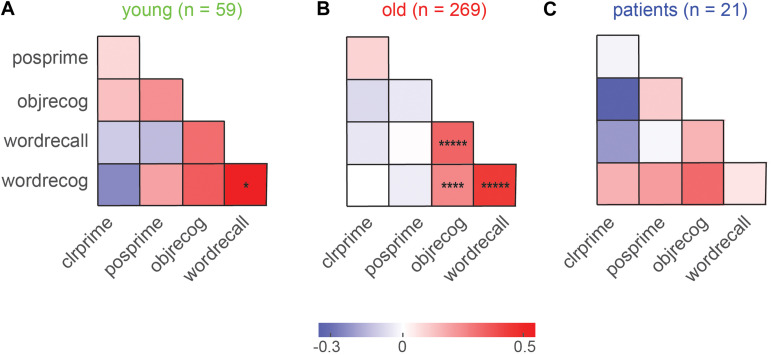
Covariation in explicit and implicit memory across participants. **(A)** Pairwise correlation between explicit and implicit memory measures for young participants. Legends: *clrprime*: color priming strength, *posprime*: position priming strength, *objrecog*: object recognition accuracy, *wordrecall*: word recall accuracy, *wordrecog*: word recognition accuracy. Each entry indicates the Pearson’s correlation coefficient between a given pair of memory measures across participants. Asterisks indicate statistical significance of these correlations (Conventions as before). **(B)** Same as **(A)**, but for older participants. **(C)** Same as **(A)**, but for MCI patients.

To investigate whether explicit and implicit memory covaried across participants, we calculated the pairwise correlation between implicit and explicit measures for each group. The resulting correlations are shown in Figure 4. We observed significant correlations only in older participants (Figure 4B), presumably because this was the group with the largest numbers. In this group, explicit memory measures were all highly correlated (Figure 4B). This is an interesting finding because the object recognition task involved simultaneously presented items with no control on item duration, whereas the word recall and recognition tasks involved items presented for fixed durations. The presence of a positive correlation implies that all three tasks are presumably governed by common explicit memory mechanisms.

By contrast, color and position priming strength were not significantly correlated (r = 0.08, *p* = 0.22; Figure 4B). Importantly, there was no significant correlation between explicit and implicit memory measures in both groups (Figures 4A,B).

We conclude that implicit and explicit memory show no covariation across participants.

### Are Explicit and Implicit Memory Measures Consistent Across Follow-ups?

Since the older participants were participants in a longitudinal study, we were able to additionally assess whether participants showed reliable implicit and explicit memory scores across years. To do we simply asked whether the measures obtained in the first year (F0) were correlated with the same measures in the subsequent follow-up visit that occurred after about a year (F1). This revealed significant correlations for all measures (r = 0.44 and 0.50, *p* < 0.005 for color and position priming, respectively; r = 0.73 and 0.67, *p* < 0.00005 for word recall and recognition, r = 0.56, *p* < 0.00005 for object recognition; all correlations across 51 participants with F0 and F1 data). Thus, both explicit and implicit memory measures have a stable signature across longitudinal follow-ups.

## Discussion

Here we characterized implicit memory using perceptual priming and explicit memory across age and cognitive impairments. Our main findings are: (1) Explicit memory was weaker in old compared to young participants, and in MCI patients compared to age- and education-matched controls; (2) Surprisingly, perceptual priming did not always decline with age: color priming was smaller in older participants but position priming was larger; (3) Position priming was less frequent in the MCI group compared to matched controls; (4) Perceptual priming and explicit memory were uncorrelated across participants. These conclusions are based on cross-sectional comparisons, so they are consistent with (but do not directly prove) a longitudinal progression of priming with age or cognitive impairment. For instance, the differences could be due to cultural or generational factors. Below we discuss these findings in the context of the existing literature.

We have found that color priming is weaker in older participants. This is consistent with a decline in repetition priming observed previously in perceptual tasks (Soldan et al., 2009; Gordon et al., 2013; Caballero et al., 2018). Our findings are unlikely to be simply due to a decline in color discrimination with age (Paramei, 2012; Schneck et al., 2014), for several reasons. First, our task requires reporting the shape of an oddball target, although it does involve identifying the oddball by its color. Second, any participant impaired in color vision would show impaired performance on the task, but in fact both young and old groups were highly accurate on this task with very few exceptions (number of participants with below-80% accuracy: 1 of 59 for young; 2 of 269 for old; even these participants had accuracy > 65%). Third, even if such participants took longer to respond, their priming strength will remain unchanged since it is a normalized measure. Fourth, among the older participants (on whom a standard color blindness test was performed), only 2 were color blind. Even these two color blind participants performed our priming task at above 95% correct, presumably because they could use luminance information alone to find the oddball target. Finally, both color discrimination and color priming might be influenced by common cognitive factors such as attention that themselves decline with age or cognitive impairments (Fernandez-Duque and Black, 2006; McDonough et al., 2019). A deeper understanding of these issues will require comparing all these properties across age and cognitive impairments.

Our finding that position priming is stronger in older participants is novel and noteworthy since most cognitive measures decline with age. What could be the underlying mechanism? One possibility is that position priming is stronger in older participants because of their longer response times (Berry et al., 2017). However, our measure of priming is normalized to each participants baseline, so it is unlikely to be affected. Moreover the longer viewing times should have led to increased color priming, but we observed the opposite pattern. Alternatively, we propose that position priming, which is the facilitation of a previously viewed position, might require overcoming a competing mechanism, namely inhibition of return (IOR) by which recently viewed locations are suppressed (Posner, 2016). Whether this is really the case remains to be established since IOR is not known to operate across time intervals spanning ∼2 s across consecutive trials such as in our study. It is also well known that inhibitory control reduces with age (Lawrence et al., 2018). We therefore propose that the loss of inhibitory control leads to decreased inhibition of return, making it easier to orient to recently detected stimuli, in turn resulting in increased position priming with age. We have also found that position priming was less prevalent among MCI participants, suggesting that it can be a useful indicator of pathological aging. Thus, our findings can be explained if both the facilitation and inhibition of previously viewed locations are differentially affected by aging and cognitive impairments. These possibilities will require further study.

Here we tested participants from an urban, highly literate background, who were all familiar with the objects and words being used for the explicit memory tests. However, participants unfamiliar with the objects or words tested could easily show biased explicit memory measures due to novelty effects (Schacter et al., 1993). This is particularly important in the Indian context and perhaps even more so in clinical settings, where participants come from a wide variety of backgrounds (urban/rural and literate/illiterate), with heterogeneous levels of visual and verbal experience. These issues can be addressed by developing standardized measures for the Indian context (Iyer et al., 2020), and by tracking cognitive measures longitudinally.

Finally, we have found that perceptual priming is uncorrelated with explicit memory across individuals. This lack of correlation between implicit and explicit memory could be due to differences in stimulus type or modality (Schacter et al., 1993; Constantinidou and Baker, 2002), nature of the task or response (Fleischman and Gabrieli, 1998; Park et al., 1998; Mitchell and Bruss, 2003; Fleischman et al., 2005; Martins and Lloyd-Jones, 2006; Gong et al., 2010; Deason et al., 2015), or because of differently probing a common memory mechanism (Berry et al., 2008; Reber, 2013; Ward et al., 2013). Alternatively, explicit and implicit memory may be dissociable (Gazzaniga et al., 2014). We therefore propose that characterizing both explicit and implicit memory measures in studies of aging or cognitive impairment can yield a deeper understanding of memory systems than measuring either measure alone.

## Data Availability Statement

All data required to reproduce the results will be made available upon request.

## Ethics Statement

The studies involving human participants were reviewed and approved by Institutional Human Ethics Committee, Indian Institute of Science, Bangalore. The patients/participants provided their written informed consent to participate in this study.

## Author Contributions

KZ, SS, AM, and SPA designed experiments. KZ and SS collected data. RG, SP, and NR coordinated the cognitive testing of older participants. KZ and SPA wrote the manuscript with inputs from all other authors. All authors contributed to the article and approved the submitted version.

## Conflict of Interest

The authors declare that the research was conducted in the absence of any commercial or financial relationships that could be construed as a potential conflict of interest.
